# Intestine-Specific Ferroportin Ablation Rescues from Systemic Iron Overload in Mice

**DOI:** 10.3390/nu18020352

**Published:** 2026-01-22

**Authors:** Cristina Castillo, Sharon Gim, Nupur K. Das

**Affiliations:** Department of Molecular and Integrative Physiology, University of Michigan, Ann Arbor, MI 48109, USA; castcris@umich.edu (C.C.); sharonbkim0708@gmail.com (S.G.)

**Keywords:** iron disorders, intestinal iron absorption, hepcidin, ferroportin, hepcidin–ferroportin axis, hemochromatosis

## Abstract

**Background/Objectives**: The hepcidin–ferroportin (Fpn1) axis is central to intestinal iron absorption, and dysregulation of this axis underlies all known forms of iron disorders. Hemochromatosis, the most common iron overload disorder in humans, results from systemic iron accumulation due to decades of uncontrolled intestinal absorption. Despite major advances in medicine in recent years, strategies for iron overload management are still lagging as they primarily rely on iron chelation and repeated phlebotomies. Fpn1, the cellular iron exporter, is ubiquitously expressed and plays a critical role in maintaining systemic iron homeostasis. **Methods**: To investigate the specific contribution of intestinal Fpn1 to systemic iron overload, we employed a CRISPR-based adenoviral hepcidin knockout mediated mouse iron overload model, combined with intestine-specific deletion of Fpn1. **Results**: An initial time-dependent experiment establishes the efficiency of hepcidin knockout (KO) by as early as 1 week of adenovirus injection. At 2 weeks of injection, a perfect reciprocal relationship between hepcidin gene suppression and liver iron levels (5–7-fold induction from the baseline) was established. Finally, intestine-specific Fpn1 deletion effectively prevented iron accumulation in hepcidin KO mice, as evidenced by nearly 4-fold lower liver iron levels compared to hepcidin KO animals with intact intestinal Fpn1. **Conclusions**: In summary, our results demonstrate that ablation of intestinal Fpn1 is sufficient to attenuate systemic iron accumulation in this mouse model of hemochromatosis. These findings suggest that selective targeting of intestinal Fpn1 may represent a promising strategy for the management of iron overload.

## 1. Introduction

Iron is essential for nearly all life forms, as it participates in numerous biochemical processes, including oxygen transport, oxidative metabolism, and DNA synthesis [1,2,3]. Due to its unique redox properties, both iron deficiency and excess are detrimental to health, and iron-related disorders represent a significant global disease burden. Systemic iron balance is maintained through three interconnected processes: (i) absorption, (ii) storage, and (iii) recycling [4].

**Figure 1:**
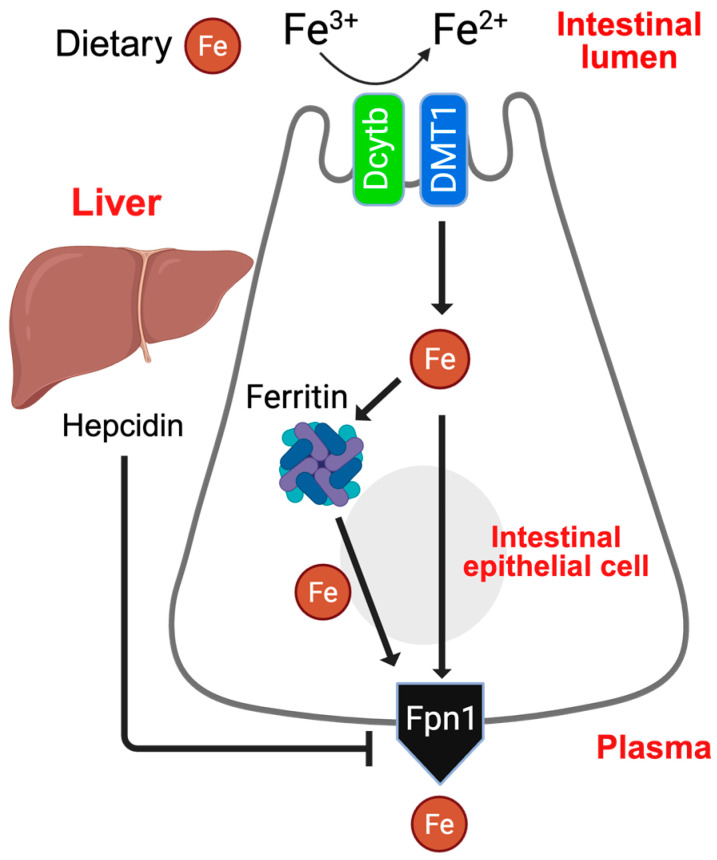
Intestinal iron absorption and its regulation by the hepcidin-Fpn1 axis. Schematic depicting the sequential steps of dietary iron absorption by intestinal epithelial cells: (i) iron influx by apical transporters, Dcytb and DMT1, and (ii) systemic delivery by the basolateral transporter Fpn1. Intestinal iron absorption is systemically maintained by the hepcidin–Fpn1 axis, where hepatic hormone hepcidin negatively regulates basolateral Fpn1 expression and activity.

Intestinal absorption of dietary iron is mediated by apical transporters, including divalent metal transporter 1 (DMT1) and duodenal cytochrome b ferric reductase (Dcytb), as well as the basolateral exporter ferroportin 1 (Fpn1). Ferritin, the cellular iron storage protein, provides a reliable iron reservoir during deficiency or high demand and serves as a major buffer against iron-induced toxicity (Figure 1). Intestinal iron absorption is regulated by two interdependent pathways: (i) local regulation via hypoxia-inducible factor (HIF)-2α-dependent transport [5] and (ii) systemic regulation via the hepcidin–Fpn1 axis, in which hepatic hepcidin limits intestinal iron efflux by inducing Fpn1 degradation [6] (Figure 1). Dysregulation of the hepcidin–Fpn1 axis is implicated in nearly all known iron-related disorders [7,8]. In iron overload, the primary defect is excessive iron export from intestinal epithelial cells, due to persistently high Fpn1 activity [7,9].

Systemic iron overload most commonly arises from genetic defects that reduce hepcidin production or impair hepcidin–Fpn1 signaling, resulting in chronically increased intestinal iron absorption and progressive parenchymal iron deposition [10,11]. Hereditary hemochromatosis (HH) is among the most common inherited metabolic disorders in individuals of Northern European ancestry. In addition to mutations in the HFE and Fpn1 genes, HH is also caused by defects in transferrin receptor 2 (TfR2), hepcidin, and hemojuvelin (HJV) [10]. If left untreated, it can lead to cirrhosis, hepatocellular carcinoma, diabetes, arthropathy, and cardiomyopathy [10,11,12,13].

Current therapies for iron overload are largely reductive, rather than disease-modifying. Therapeutic phlebotomy remains the standard of care and is highly effective at reducing the total body iron stores. In contrast, iron chelation is generally reserved for patients who cannot tolerate or access phlebotomy [12,14,15]. However, these approaches require frequent clinical visits, which are often associated with adverse effects and rely heavily on long-term adherence [12,15,16]. These limitations have motivated an increasing interest in strategies that directly target the hepcidin–Fpn1 axis, including hepcidin mimetics and ferroportin inhibitors, to reduce pathological iron flux, rather than repeatedly removing accumulated iron [13,14,17].

Fpn1 is expressed not only in intestinal epithelial cells, but also ubiquitously, including in macrophages and hepatocytes, underscoring its central role in systemic iron homeostasis [11,12]. Despite extensive characterization of the hepcidin–Fpn1 axis, the specific contribution of intestinal Fpn1 to systemic iron overload under conditions of hepcidin deficiency remains incompletely defined. Addressing this gap is essential for evaluating intestine-centered therapeutic strategies that modulate dietary iron entry without globally perturbing iron recycling pathways.

Here, we directly test this hypothesis, using a Cre-independent hepcidin knockout model combined with inducible, intestine-specific ablation of Fpn1 to define the functional requirement for intestinal Fpn1 in the development of systemic iron overload.

## 2. Materials and Methods

### 2.1. Animals and Treatments

All mice used in this study were on a C57BL/6J background. Mice were housed in a temperature-controlled (21 ± 1.1 °C), specific-pathogen-free (SPF) facility on a 12 h light/dark cycle and fed ad libitum with a standard chow diet (5L0D; PicoLab^®^ Laboratory Rodent Diet, Fort Worth, TX, USA; www.labdiet.com) containing 240 ppm iron. Here is a brief list of key feature/ingredients of the chow diet: total digestible nutrients 73%; gross energy 4.08 kcal/gm; physiological fuel value 3.35 kcal/gm; protein 24.1%; fat (ether extract) 5.1%; zinc 75 ppm; manganese 71 ppm; copper 13 ppm; iodine 1 ppm; and selenium 0.41 ppm. Both male and female mice were used throughout, and littermates were randomly assigned to experimental groups. For time-dependent analysis of hepcidin knockout, 5 time points (0, 1, 2, 4, and 8 weeks; *n* = 4 per group) were assessed with an equal number of male and female mice. Tamoxifen-inducible, intestine-specific *Fpn1* KO mice (Fpn1^ΔIE^:Vil^CreERT2^; Fpn^fl/fl^) have been described [18]. For the experiment related to the combination of intestinal *Fpn1* ablation with hepcidin knockout, Fpn^fl/fl^ (Cre negative) and Fpn1^ΔIE^:Vil^CreERT2^ (Cre positive) mice were treated or untreated with hepcidin-targeting adenovirus (total of 4 groups; *n* = 4 per group) with equal distribution of male and female mice. Mucosal scrapings of the duodenum to isolate enterocytes were used in all experiments.

### 2.2. CRISPR-Mediated Hepcidin-KO Model

For hepcidin ablation in mice, two guide RNAs targeting mouse hepcidin were cloned in a CRISPR-Cas9-containing adenovirus dual gRNA expression vector (pAV[CRISPR]-hCAS9; VectorBuilder, Inc., Chicago, IL, USA). Hepcidin-targeting CRISPR-Cas9-containing adenovirus (AV) was prepared by Vector Core, University of Michigan Medical School, at a concentration of 4 × 10^12^ particles per mL. Mice were treated with AV, as previously described [19]. Briefly, the stock AV solution was diluted in sterile 0.9% NaCl solution, and the volume was adjusted to 150 µL for delivery of 3 × 10^11^ particles per animal via tail vein injection. AV injection and post-injection mice maintenance were performed in the Containment Facility Room in the Unit for Laboratory Animal Medicine (ULAM), University of Michigan.

### 2.3. Antibodies

Anti-rabbit ferritin H (Cell Signaling Technology [CST], Danvers, MA, USA; Catalog# 3998S; Lot 2); anti-rabbit ferroportin 1 (ADI, San Antonio, TX, USA; Catalog# MTP11-A; Rabbit Anti-Mouse MTP1 IgG # 1 (aff pure)); anti-mouse actin, (1:10,000; Proteintech, Rosemont, IL, USA; Catalog# 66009-1-Ig; Lot 10052031). Dilutions were 1:1000, unless otherwise specified.

### 2.4. Real-Time Quantitative PCR

A total of 1 μg of total RNA extracted using trizol reagent (ThermoFisher Scientific, Ann Arbor, MI, USA; Catalog# 15596018) from mouse duodenal epithelial scrapes were reverse-transcribed to cDNA, using the SuperScript™ III First-Strand Synthesis System (ThermoFisher Scientific, Ann Arbor, MI, USA; Catalog# 18080051). Quantitative PCR (qPCR) reactions were set up in three technical replicates for each sample with SYBR green master mix (ThermoFisher Scientific, Ann Arbor, MI, USA; Catalog# A25918) and run in QuantStudio 5 Real-Time PCR System (Applied BioSystems, Waltham, MA, USA). Gene expression levels (fold-change) were analyzed by using the ΔΔCt method, with β-actin as the housekeeping gene. The following primers were used: DMT1: forward, 5′-TGTTTGATTGCATTGGGTCTG-3′; reverse, 5′-CGCTCAGCAGGACTTTCGAG-3′. Dcytb: forward, 5′-CATCCTCGCCATCATCTC-3′; reverse, 5′-GGCATTGCCTCCATTTAGCTG-3′. Fpn1: forward, 5′-ATGGGAACTGTGGCCTTCAC-3′; reverse, 5′-TCCAGGCATGAATACGGAGA-3′. Hepcidin: forward, 5′-CTATCTCCATCAACAGATGAGACAGA-3′; reverse, 5′-AACAGATACCA CACTGGGAA-3′. β-actin: forward, 5′-TGAAGCAGGCATCTGAGGG -3′; reverse, 5′-CGAAGGTGG AAGAGTGGGAG-3′. The sequence accuracy of all the primers was verified by in silico PCR by using Primer Blast, NCBI. Functionally, the primers were verified by the relevant gene expression analyses of appropriate mouse models, as described in our previous works [19,20].

### 2.5. Western Blotting

Whole-cell and membrane lysate preparations were described previously [20,21]. For ferritin heavy chain (Ftn H) Westerns, whole-cell lysates were prepared from duodenal scrapes. Briefly, tissue homogenates were incubated in RIPA buffer (supplemented with protease inhibitor cocktail) on ice for 15–20 min, followed by centrifugation at 12,000 rpm for 20 min at 4 °C. Supernatants were transferred to a new tube, mixed with 5X Laemmli buffer, and boiled for 5 min. For ferroportin 1 (Fpn1) Westerns, membrane lysate was prepared by dounce homogenization (~25 strokes) of duodenal scrapes, followed by passing the samples through a 27-gauge needle 10–15 times in lysis buffer (Tris pH 8.0, 5 nM; EDTA, 2 mM; sucrose, 250 mM; and protease inhibitor). The nuclei were first pelleted at 1000× *g* for 10 min, followed by supernatant centrifugation at 45,000 rpm for 45 min using Optima™ TLX Ultracentrifuge (Beckman Coulter Life Sciences, Brea, CA, USA). The resulting membrane pellet was resuspended in storage buffer (Tris 75 mM pH 8.0; MgCl_2_ 12.5 mM; EDTA 5 mM; and protease inhibitor). Membrane extracts were mixed with 2X Laemmli buffer and incubated at 40 °C for 5 min. Ponceau staining was used for verifying the equal loading of the membrane extracts, as described in our previous works [18,19,20].

### 2.6. Tissue Iron Analysis by ICP-MS (Inductively Coupled Plasma Mass Spectrometry)

Mouse livers and duodenal scrapes were digested with 2 mL/g total wet weight nitric acid (Trace metal grade; ThermoFisher Scientific, Ann Arbor, MI, USA) for 16 h (RT), followed by treatment with 1 mL/g total wet weight hydrogen peroxide (Trace metal grade; ThermoFisher Scientific, Ann Arbor, MI, USA;) for another 16 h (RT). Samples were diluted with ultrapure water (VWR Chemicals ARISTAR^®^ ULTRA, Allentown, PA, USA), followed by ICP-MS, (Nexion 2000, Perkin Elmer, Shelton, CT, USA) using 50 ppb Bismuth as an internal standard.

### 2.7. Quantification and Statistical Analysis

The data distribution for the controls and the corresponding experimental group(s) have similar variances. Therefore, no sample or data were excluded from the study for statistical purposes. Normality tests were performed for the gene expression studies. Statistical details for all experiments are provided in the figure legends. Results are expressed as the mean ± SEM, because compared to standard deviation (STD), standard error is more useful as a means for calculating the confidence interval [22]. The significance between 2 groups (control vs. treatment) was tested using a 2-tailed, unpaired *t* test. The significance among multiple groups (to compare means of three or more groups) was tested using a one-way ANOVA, followed by Tukey’s post hoc test. GraphPad Prism 10.0 was used to conduct the statistical analyses. The statistical significance is described in the figure legends as follows: ** *p* < 0.01, *** *p* < 0.001, and **** *p* < 0.0001.

## 3. Results

### 3.1. A Cre-Independent Iron Overload Model via Adenovirus-Mediated Rapid and Durable Hepcidin KO

**Figure 2:**
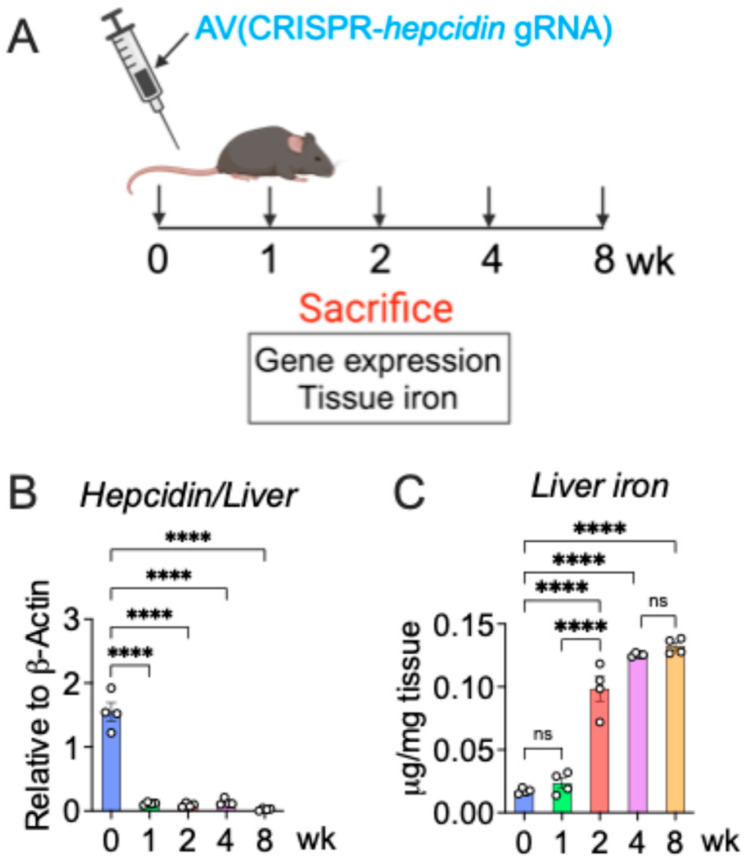
CRISPR-based mouse model of hemochromatosis. (**A**) Schematic of the development of CRISPR-based hepcidin KO mouse model by tail vein injection of adenovirus (AV) at various time points; (**B**) hepatic hepcidin gene expression (*n* = 4 per group); (**C**) liver iron analysis (*n* = 4 per group). Mice were 4–6 weeks old. All data are mean ± SEM. One-way ANOVA with Tukey’s multiple comparisons test (for (**B**,**C**)). **** *p* < 0.0001; ns: not significant.

We previously developed a robust mouse model of iron overload using a CRISPR/Cas9-based, adenovirus (AV)-mediated hepcidin knockout approach (AV-*Hepc* KO) [19]. A time-course analysis following a single AV injection was conducted to assess the onset and durability of hepcidin suppression. Hepatic hepcidin expression was significantly reduced at 1, 2, 4, and 8 weeks post-administration, demonstrating durable, on-target activity in vivo (Figure 2A). To evaluate the earliest tissue-level effects of hepcidin knockout, we measured the hepatic iron content over the same time points. While no significant change was observed at week 1, liver iron levels increased dramatically by week 2 and remained elevated throughout the study period (Figure 2B). Collectively, these results indicate that AV-Hepc KO induces rapid and sustained hepcidin suppression, leading to robust systemic iron overload as early as 2 weeks post-injection.

### 3.2. AV-Hepc KO Activates the Enterocyte Iron Transport Machinery

**Figure 3:**
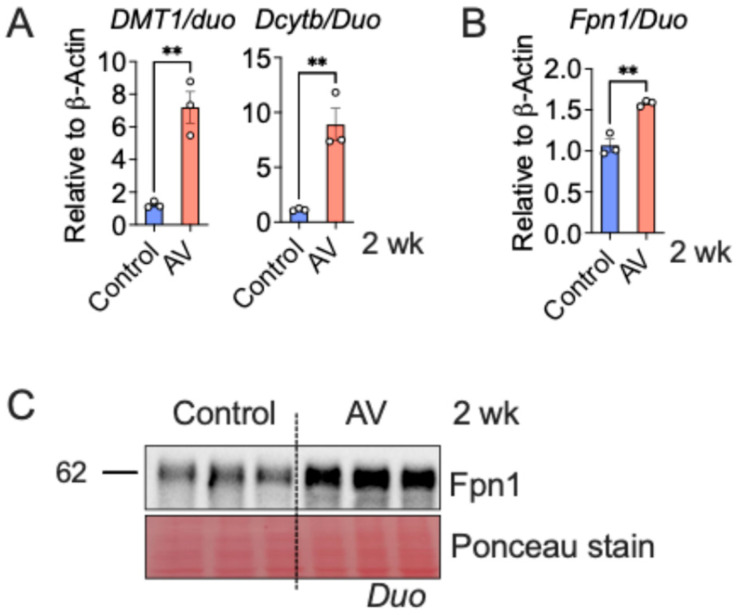
Adenovirus (AV)-mediated Hepcidin KO upregulates the expression of intestinal iron absorptive genes. Mice were treated with AV via tail vein injection, followed by gene expression analysis of apical iron transporters, i.e., *DMT1* and *Dcytb* (**A**), basolateral iron exporter, i.e., *Fpn1* (**B**); and Fpn1 Western analysis (**C**) in the duodenum (*n* = 3 per group). Mice were 4–6 weeks old. All data are mean ± SEM. Unpaired *t* test (for (**A**,**B**)). ** *p* < 0.01.

Next, we analyzed intestinal transporter gene expression at the 2 week time point following AV delivery. AV-mediated hepcidin knockout led to significant increases in the expression of DMT1 and Dcytb (apical iron uptake) (Figure 3A) and Fpn1 (basolateral iron export) (Figure 3B) compared to controls. These changes are consistent with a low-hepcidin state that enhances Fpn1-dependent iron transport from the intestinal lumen into systemic circulation. Western blot analysis confirmed these findings, showing elevated Fpn1 protein levels in AV-treated mice (Figure 3C).

### 3.3. AV-Hepc KO Leads to Enterocyte Iron Depletion

**Figure 4:**
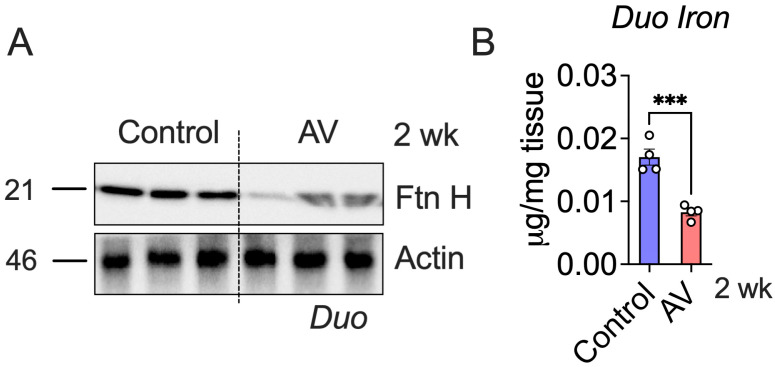
Adenovirus (AV)-mediated Hepcidin KO causes iron depletion in duodenal enterocytes. Mice were treated with AV via tail vein injection, followed by ferritin heavy chain (Ftn H) Western analysis (**A**); (*n* = 3 per group), and iron level quantification (**B**) in the duodenum; (*n* = 4 per group). Mice were 4–6 weeks old. All data are mean ± SEM. Unpaired *t* test (for (**B**)). *** *p* < 0.001.

In AV-Hepc KO mice, hepcidin deletion-mediated intestinal hyperabsorption [18,19] led to a significant decrease in duodenal enterocyte iron levels compared to the controls (Figure 4A). Consistent with this observation, levels of the iron storage protein ferritin-H (Ftn H) were markedly reduced in AV-treated duodena (Figure 4B), indicating enhanced iron flux through enterocytes in our hepcidin KO model. Together with the data in Figure 2, which show upregulation of DMT1, Dcytb, and Fpn1, these results suggest that AV-Hepc KO induces a sustained cellular iron demand, met through a combination of increased apical iron uptake and persistent depletion of intracellular iron stores, provided that Fpn1 is present and accessible at the cell surface.

### 3.4. Intestine-Specific Fpn1 Ablation Prevents Hepc KO-Mediated Iron Overload

**Figure 5:**
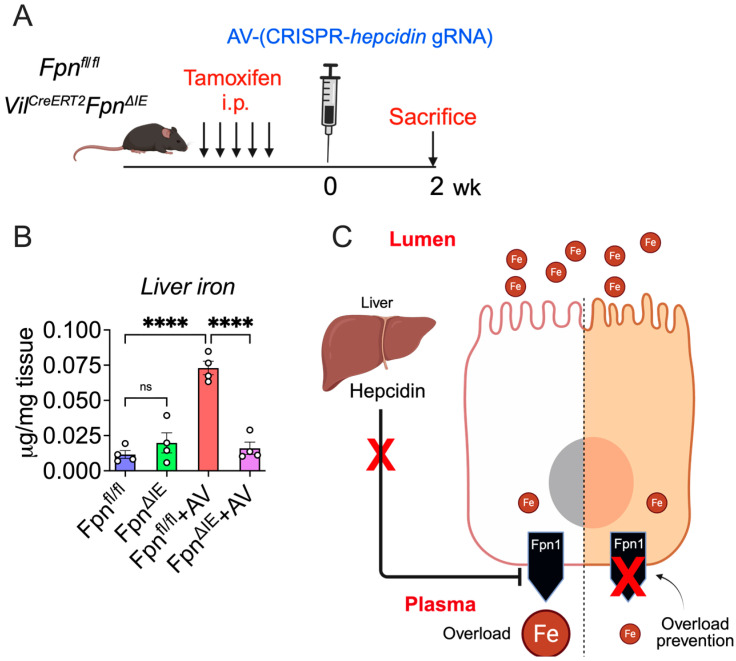
Intestine-specific *Fpn1* ablation protects mice from systemic iron overload. (**A**) Schematic of AV treatment in Fpn1^ΔIE^ and wild-type littermates; (**B**) liver iron analysis (*n* = 4 per group); and (**C**) schematic depicting the critical role of intestinal Fpn1 in hepcidin KO-mediated systemic iron overload. The schematic highlights that in the absence of hepcidin, intestinal ferroportin is required to sustain dietary iron flux into the circulation and drive systemic iron overload. Mice were 4–6 weeks old. All data are mean ± SEM. One-way ANOVA with Tukey’s multiple comparisons test (for (**B**)). **** *p* < 0.0001; ns: not significant.

To determine the protective role of intestinal Fpn1 deletion against systemic iron overload, we used tamoxifen-inducible, intestine-specific *Fpn1* KO mice (Fpn^ΔIE^) in combination with AV-mediated hepcidin KO. Briefly, Vil^CreERT2^; Fpn^fl/fl^ littermate control mice were (a) administered five doses of tamoxifen, followed by (b) a single AV injection seven days after the final tamoxifen dose, and were (c) sacrificed two weeks after AV treatment (Figure 5A). In the control mice, (Fpn^fl/fl^) liver iron levels increased significantly following AV injection, which was consistent with our initial experiments (Figure 2C and Figure 5B). In contrast, liver iron levels in the double KO mice returned to baseline levels, highlighting the essential role of intestinal Fpn1 in the development of iron overload (Figure 5B). The schematic summary (Figure 5C) illustrates this mechanism: hepcidin loss allows Fpn1-mediated iron mobilization, but intestinal Fpn1 is required to sustain iron flux from the diet to the plasma. These data demonstrate that intestine-specific Fpn1 ablation can effectively prevent systemic iron overload. Our finding serves as a proof-of-concept study, showing that intestine-specific Fpn1 inhibition can offer a stand-alone or adjunct therapy for systemic iron overload disorders. Interestingly, vamifeport, an oral Fpn1 inhibitor, has shown considerable efficacy in both primary and secondary iron overload by effectively reducing intestinal iron absorption [23,24].

## 4. Discussion

Hepcidin is the master regulator of systemic iron homeostasis, acting by binding to Fpn1 and inducing its internalization and degradation [11,17,25,26]. Although Fpn1 is expressed in multiple iron-donor cell types, including macrophages, hepatocytes, and enterocytes, foundational studies have demonstrated that intestinal Fpn1 plays a particularly critical role in systemic iron regulation [27,28,29,30,31]. However, it remained unclear whether selectively limiting intestinal Fpn1 activity could provide a therapeutic advantage in disorders that are characterized by intestinal iron hyperabsorption. Prior works by Schwartz and colleagues provided key mechanistic insights into the intestinal contribution to systemic iron regulation. They defined a functional hepatic hepcidin–intestinal HIF-2α axis that coordinates dietary iron absorption across both iron-deficient and iron-overload states, establishing that enterocyte transcriptional programs actively shape systemic iron flux, rather than simply responding passively to the circulating iron levels [20]. Due to the persistent lack of iron efflux at the basolateral membrane, intestine-specific Fpn1 knockout (Fpn1^ΔIE^) mice develop severe iron deficiency anemia (IDA), while the enterocytes become overloaded due to dietary iron retention [11]. Consequently, in response to continued systemic iron deficiency, the cells (e.g., hepatocytes and macrophages) with intact Fpn1 activity continue to export iron into the systemic circulation and become iron-depleted [27]. Complementing this, another study demonstrated that long term intestinal Fpn1 deficiency eventually (within 4–5 months of inducible deletion) leads to end-stage IDA, tissue-specific transcriptional stress responses that cause inflammatory damage to the liver parenchyma, and cardiomegaly [28]. In the context of colorectal cancer (CRC), colon-specific Fpn1 deletion demonstrated that Fpn1 deficiency mediated iron trapping within the CRC epithelium, thereby enhancing CRC pathogenesis [32]. Additionally, Fpn1 is also expressed in the colonic macrophages and neutrophils, which are amenable to regulation by dendritic cell (DC)-derived hepcidin. Dysregulation of the hepcidin–Fpn1 axis in the colonic macrophages and neutrophils favors colonic inflammation and impairs mucosal healing [33]. In humans, dysregulation of the hepcidin–Fpn1 axis is a central pathogenic mechanism underlying iron overload disorders. Inappropriately low hepcidin production or impaired hepcidin responsiveness results in sustained Fpn1 activity at the basolateral membrane of enterocytes, leading to excessive dietary iron absorption, elevated transferrin saturation, and progressive parenchymal iron loading in the liver and other organs. Consistent with this paradigm, genetic and acquired defects that blunt hepcidin signaling or prevent effective Fpn1 downregulation phenocopy classical hemochromatosis by promoting unrestrained intestinal iron export into the systemic circulation. These clinical observations reinforce Fpn1’s pivotal role as the final common effector of systemic iron homeostasis and provide a strong biological rationale for therapeutic strategies that selectively limit intestinal Fpn1 activity to mitigate pathological iron hyperabsorption. Together, these experimental and clinical observations underscore the intestine as a dominant regulatory site where iron transport programs integrate local and systemic cues. Importantly, while these studies established how systemic and local signals transcriptionally regulate intestinal iron transport programs, they did not directly test whether intestinal Fpn1 activity is required for systemic iron overload to occur in vivo.

Our study extends these foundational observations by providing direct genetic evidence that intestinal Fpn1 is not only regulated by hepcidin and HIF-2α-dependent pathways [6,20,30] but is also functionally required for iron overload to develop in vivo. By genetically uncoupling intestinal iron efflux from systemic hepcidin deficiency, our data establish a causal link between enterocyte Fpn1 activity and peripheral tissue iron accumulation.

Clinically, systemic iron overload disorders are managed by a systemic iron depletion strategy via intermittent phlebotomy and iron chelation [34]. These are associated with major adverse effects, low treatment adherence, and high cost [35,36]. Persistent iron hyperabsorption points towards the necessity for novel approaches directly targeting the hepcidin–Fpn1 axis in the forms of hepcidin replacement [37,38] (e.g., minihepcidin [39,40], hepcidin agonists [41], or mimetics [42]) or Fpn1 inhibition [43]. Interestingly, in animal models of hepcidin deficiency, exogenous hepcidin effectively reduced intestinal iron absorption but failed to mobilize excess liver iron [34]. Our study demonstrates that direct targeting of enterocyte iron efflux via Fpn1 ablation is highly efficacious in preventing iron overload (Figure 5B) which, in humans, is anticipated to significantly reduce the need for maintenance phlebotomy and iron chelation.

Building on this framework, our AV-Hepc KO model induces robust hepatic hepcidin suppression without reliance on tissue-restricted Cre drivers, thereby avoiding confounding effects associated with Cre-mediated alterations in either hepatocytes or enterocytes alone. In this low-hepcidin state, we observe the expected coordinated intestinal response, including upregulation of the apical iron uptake machinery (DMT1 and Dcytb) and increased basolateral Fpn1 expression, recapitulating the pathophysiologic mechanisms observed in hereditary hemochromatosis and related disorders [13,14,15,16,44]. Consistently, duodenal iron content and ferritin-H levels were depleted, confirming enhanced mucosal iron flux. Importantly, despite this strong intestinal activation, systemic iron elevation remained critically dependent on intestinal Fpn1 (Figure 5B). Although this data represents a rather short-term intervention, we anticipate that a long-term, sustained intestinal Fpn1 deficiency would continue to prevent the buildup of systemic iron overload mediated by hepcidin knockout. While macrophage Fpn1 is essential for iron recycling [17,25], these stores cannot compensate for the absence of intestinal export when hepcidin is suppressed. Together, these findings indicate that intestinal Fpn1 is the primary driver of systemic iron regulation. These findings demonstrate that intestinal Fpn1 is necessary for translating hepcidin deficiency into systemic iron accumulation.

Recently, vamifeport, an orally bioavailable small-molecule Fpn1 inhibitor that directly binds with Fpn1 and competes with hepcidin to inhibit Fpn1-mediated iron export, has entered clinical development based on promising preclinical efficacy in disorders of ineffective erythropoiesis and iron overload [36,37,38]. Preclinical studies demonstrate that vamifeport reduces dietary iron absorption and prevents tissue from iron-loading in models of hemochromatosis, indicating substantial activity at the level of enterocyte ferroportin [36]. However, its action is not intestine-specific and is most effective in disorders associated with ineffective erythropoiesis (e.g., thalassemia and sickle cell disease), where global Fpn1 inhibition provides a clear therapeutic benefit [37,38]. Together, these observations underscore that a substantial component of vamifeport’s efficacy derives from limiting dietary iron entry at the level of enterocyte Fpn1, providing important pharmacologic context for our genetic findings that demonstrate that selective disruption of intestinal Fpn1 is sufficient to prevent systemic iron overload. In this context, generation of intestine-specific Fpn1 inhibitors either through vamiferport modification or novel chemical space development would be critical for targeting intestinal hyperabsorption in human iron overload disorders. However, challenging factors such as pH, transit times, enzymes, and complex gut anatomy preclude the development of agents with site-specific efficacy.

Conversely, these insights are highly relevant for iron-restricted anemias. Conditions such as anemia of chronic inflammation, malignancy, or chronic kidney disease (CKD) are characterized by elevated hepcidin, making them potential candidates for therapeutic restoration of intestinal Fpn1 activity through hepcidin-lowering strategies [45,46,47,48,49]. Individuals with ferroportin disease caused by loss-of-function SLC40A1 variants may have reduced or absent enterocyte Fpn1 and may not respond adequately to hepcidin suppression, despite intact macrophage or hepatic Fpn1 [14,16,44]. These results highlight the importance of assessing the intestinal Fpn1 status when selecting patients for hepcidin-modulating therapies.

The modular design of the AV platform described here also enables bidirectional therapeutic strategies. Importantly, this approach allows for controlled modulation of the hepcidin–Fpn1 axis without relying on tissue-restricted Cre drivers. While the current study focuses on direct ablation of intestinal Fpn1 to treat iron overload, AV-mediated hepcidin overexpression could be applied to thalassemic or transfusional iron overload. Elevating hepcidin would reduce intestinal Fpn1, limit dietary iron absorption, and promote safe macrophage sequestration, which is consistent with the previously reported benefits of hepcidin agonists and mimetics in preclinical models [17,39]. Thus, a single gene therapy platform could potentially address both ends of the iron-dysregulation spectrum.

Although our work establishes the precise mechanistic role of intestinal Fpn1 in the pathogenesis of systemic iron overload, this study has a few limitations: (i) Intestine-specific Fpn1 ablation prior to hepcidin suppression implies its preventive role in iron overload development. However, to confirm its therapeutic role, intestinal Fpn1 ablation after the development of iron overload would be necessary. (ii) The precise role of extra-intestinal Fpn1 in the current context was not explored. (iii) This study focused on the short-term outcomes of intestinal Fpn1 ablation in systemic iron overload. Exploration of long term effects would provide more definitive answers regarding both the efficacy and potential off-target consequences of the intestine-specific Fpn1 deletion. Although our study provides proof-of-concept that intestinal Fpn1 ablation has therapeutic potential for hemochromatosis therapy, pharmacological targeting of intestinal Fpn1 should be prioritized for effective disease management.

## 5. Conclusions

In summary, our findings demonstrate that intestinal Fpn1 is a decisive control point for systemic iron loading under conditions of hepcidin deficiency, and that limiting dietary iron export from the intestine is sufficient to prevent iron accumulation in peripheral tissues. By genetically uncoupling intestinal iron efflux from systemic hepcidin loss, this study provides direct in vivo evidence that enterocyte iron handling is not merely permissive but actively determines the whole-body iron balance. While the adenoviral hepcidin knockout (AV-Hepc KO) model represents a severe perturbation of hepcidin signaling and complete intestinal Fpn1 ablation may exceed the degree of inhibition achievable with pharmacologic approaches, these data nonetheless establish a clear mechanistic framework for evaluating intestine-centered strategies to modulate iron flux. Future studies comparing intestine-restricted versus systemic Fpn1 inhibition and defining the long-term consequences of partial ferroportin suppression will be essential for translating these insights into safe and effective therapies for iron overload and iron-restricted anemias.

## Data Availability

Data are contained within the article. The original contributions presented in this study are included in the article. Further inquiries can be directed to the corresponding author.

## Abbreviations

The following abbreviations are used in this manuscript:

CRISPR	Clustered Regularly Interspaced Short Palindromic Repeats
HIF	Hypoxia Inducible Factor
Ftn	Ferritin
Fpn	Ferroportin
